# Safety and efficacy of different therapeutic regimens in Egyptian adults with moderate COVID-19 infection (EVEREST): a real-world retrospective study

**DOI:** 10.1038/s41598-025-23660-1

**Published:** 2025-10-20

**Authors:** Mohamed Abdel-Salam Elgohary, Nouran Hamza, Asmaa Ali, Raafat Zaher Abdel-Rahman, Mohamed Thabet Elnagar, Mohamed Emam Mohamed, Mohamed G. Seadawy, Mahmoud Zenhom Abdelfatah, Nouran A. Taha, Marina Rauof, Maisra M. El-Bouseary

**Affiliations:** 1 Egypt Center for Research and Regenerative Medicine (ECRRM), Cairo, Egypt; 2 Military Medical Academy, Cairo, Egypt; 3 MARS-GLOBAL, London, UK; 4 Department of Pulmonary Medicine, Abbassia Chest Hospital, MOH, Cairo, Egypt; 5 Department of Laboratory Medicine, School of Medicine, Jiangsu University, Zhenjiang, 212013 People’s Republic of China; 6 Manager of Armed Forces Laboratory of Medical Research and Blood Bank (AFLMR), Cairo, Egypt; 7 Public Health Department, Military Medical Academy, Cairo, Egypt; 8 Military Medical Academy, Cairo, Egypt; 9 Biological Prevention Department, Ministry of Defense, Cairo, Egypt; 10 Department of Pharmaceutical Microbiology, Faculty of Pharmacy, Tanta University, Tanta, Egypt

**Keywords:** COVID-19, Ivermectin, Sovodak, Sofosbuvir, Ledipasvir, Hydroxychloroquine, Viral infection, Respiratory distress syndrome

## Abstract

This study seeks to disseminate insights from Egypt’s management of COVID-19 patients by evaluating the effectiveness and safety of various treatment regimens using combined repurposed antivirals. A retrospective cohort study was conducted on 310 moderate hospitalized COVID-19 cases. Patients were divided into four treatment arms: standard care, sofosbuvir/daclatasvir (sovodak) plus ivermectin, sofosbuvir/ledipasvir (SOF/LED) plus hydroxychloroquine, and SOF/LED plus ivermectin. The study analyzed parameters such as hospitalization days, total clinical recovery percentage, and progressive CT chest changes. The median hospitalization days significantly differed in arm 1 versus arms 2 and 3 (*p* < 0.001 and *p* = 0.025, respectively). There was a difference between arms 1 and 4 in the days till clinical improvement (*p* = 0.007). Complete normalization of vital signs occurred in 26% of arm 4 patients versus 43% in arm 1 (*p* = 0.023), and a statistically significant difference in the proportion of patients with total clinical recovery was found between arms 1 and 2 (*p* = 0.009). All arms displayed a statistically significant lower proportion of patients with progressive CT scans compared to arm 1. Our study reveals that most tested antiviral combinations effectively reduced hospitalization days and progressive CT scans. These regimens demonstrated efficacy in treating moderate COVID-19 to prevent disease progression and complications.

**Supplementary Information:**

The online version contains supplementary material available at 10.1038/s41598-025-23660-1.

## Introduction

 Coronavirus disease 2019 (COVID-19) is caused by severe acute respiratory syndrome coronavirus-2 (SARS-CoV-2), a positive-sense RNA virus. By the end of December 2022, Egypt had officially registered 515,645 cases of COVID-19 and 24,802 deaths, according to an analysis report published in May 2023. Comparably to other countries, Egypt reported 4646 confirmed cases and 223 deaths per million Egyptians, whereas the global average was 83,235 cases and 841 deaths per million people^1,2^ Both are considered significant undercounts by the World Health Organisation (WHO). Due to inadequate testing and difficulties in determining the cause of death, the true death toll from COVID-19 is probably higher than the number of documented deaths.

The emergence of novel strains such as delta and omicron, disparities in vaccine accessibility across nations, and varying levels of acquired immunity among individuals demonstrated that, despite the high efficacy of vaccines, the pandemic is still far from over. As a result, the demand for a therapeutic substitute persists. Repurposing the currently existing medications is one less expensive and more scalable strategy^3^. Drug repurposing has several advantages over the conventional drug development method, including offering a substantial amount of existing data from a significant number of studies, proven safety records, and decreased failure risk^4^. Additionally, one important when selecting these repurposed drugs, particularly antivirals, is that they should be easy to administer, must lack significant side effects or negative interactions with other medications, have an affordable price for low- or middle-income countries, and be easy to mass-produce, distribute, and store^5^.

One famously used repurposed drug for COVID-19 is hydroxychloroquine (HCQ). Studies have revealed that hydroxychloroquine is a potent preventive and therapeutic alternative as a repurposed drug. However, relatively few clinical trials were conducted and low recoveries were reported requiring more research on its effectiveness^3^. Another widely used treatment option is ivermectin. Previous reports have found that ivermectin exhibits broad-spectrum antiviral activities^6,7^ thus justifying its use for COVID-19. Additionally, a systematic review established that using ivermectin early in the disease’s clinical course might decrease the cases progressing to severe disease^8^.

Repurposing known antivirals has also become a popular strategy in the fight against COVID-19. The study of combination therapies, consisting of two or more direct-acting antivirals from a different class (e.g., a nucleotide analog plus monoclonal antibody), is considered the most effective method for quickly assessing the efficacy of potential COVID-19 therapies and will likely reduce the chance of escape variants emerging thus improving the clinical benefit of treatment^5,9^.For example, many ongoing clinical trials are exploring the potential of combining remdesivir (a nucleotide analog) with repurposed drugs by simultaneously targeting multiple targets of viruses and host humans^10^. Moreover, daclatasvir may act on the SARS-CoV-2 RNA polymerization complex, similar to its activity against HCV, where it could disrupt COVID-19 viral replication and reduce the severity of the infection^11^. Furthermore, the combination of daclatasvir and sofosbuvir, which has a proven safety record and enhances clinical results in hepatitis C virus (HCV)patients^12^, may have antiviral effectiveness against SARS-CoV-2, according to in vitro and in silico research^12^. Other repurposed combination antivirals include sofosbuvir/ledipasvir (SOF/LED), which is an FDA-approved drug for HCV treatment^13^. It has been tested in multiple clinical trials with promising results^14,15^. Having no serious known adverse events or related drug-drug interactions with either HCQ or ivermectin makes these antivirals a potential option for moderate COVID-19 patients.

The scope of COVID-19 studies tested different treatment strategies but did not focus much on the specific combination of antivirals plus HCQ or ivermectin in Egyptian patients. Therefore, our study aimed to assess the efficacy and safety of multiple therapeutic regimens including (Sofosbuvir, Daclatasvir, Ledipasvir, and Ivermectin) in Egyptian adults with moderate COVID-19 infection.

## Methods

### Study design and participants

This is a retrospective cohort study conducted in Almaza Fever Hospital, Cairo, Egypt, from December 2020 to December 2022.

Initially, patients were screened for fulfilling the inclusion/exclusion criteria, and finally, 310 hospitalized patients were included in our study. We collected demographic data from hospital records including age, gender, complete blood count (CBC), serum creatinine, liver function tests, D-dimer, serum ferritin, C-reactive protein (CRP), interleukin-6 (IL6), and triphasic chest computed tomography CT (evaluated by two independent radiologists for reliability, and the lesion was categorized as mild, moderate, or severe)^16^. Routine lab tests were conducted at admission and every three days, so we collected data from four different measurements at different time points.

This study followed ethical guidelines based on the Helsinki Declaration, and informed written consent was taken from each patient. Additionally, the study has been approved by the ethics committee office of the Medical Military Academy. Our study is reported following the STROBE reporting checklist.

### Inclusion and exclusion criteria

Patients were included in our study if they were: aged ≥ 18 and ≤ 75, confirmed to be positive for COVID-19 by Reverse Transcription Polymerase Chain Reaction (RT‒PCR), and met the Egyptian Ministry of Health and Population (MOHP) protocol criteria for moderate COVID-19 cases (fever “measured temperature of at least 38 °C”, lower respiratory symptoms “cough, shortness of breath”, SpO2 ≥ 92%, and imaging-confirmed pneumonia”)^17,18^.

Patients with mild, severe and critically ill COVID-19 disease were excluded; mild disease means mild symptoms without signs of hypoxia or viral pneumonia; severe disease means SpO2 < 92%, PaO2 /FiO2 < 300, respiratory rate > 30 breaths/min, or lung infiltrates > 50%; critical COVID-19 means manifestation in any one of the subsequent circumstances: (1) shock; (2) respiratory failure requiring mechanical ventilation; and (3) other organ failure requiring ICU care and severe liver disease (e.g., AST > 5 times upper limit, child Pugh score ≥ C).

### Treatment regimen

Patients were divided into 4 arms according to the received antiviral regimen; Arm 1: Subjects received the Egyptian MOHP regimens, including HCQ (400 mg/12 h on the first day followed by 200 mg /12 h for 6 days) and ivermectin (0.4 mg/kg daily for 4 days)^17^; Arm 2: Subjects received sofosbuvir 400 mg, and daclatasvir 60 mg (sovodak) once daily for 14 days plus ivermectin (0.4 mg/kg daily for 4 days); Arm 3: Subjects received one tablet containing sofosbuvir and ledipasvir (SOF/LED) (400 mg + 90 mg) once daily for 14 days plus HCQ (400 mg/12 h on the first day followed by 200 mg/12 h for 6 days); and Arm 4: Subject received 0.4 mg/kg ivermectin daily for 4 days and SOF/LED (400 mg + 90 mg) once daily for 14 days plus ivermectin (0.4 mg/kg daily for 4 days). All patients received adjuvant medications reported in our national protocol^17,18^, including immunomodulators, anti-inflammatory (dexamethasone 6 mg or its oral equivalent), anticoagulation (if the D-dimer is 500–1000, preventative; if it is 1000 or higher, therapeutic), and prophylactic β-lactam (ceftriaxone, 1 gm daily).

### Study variables/outcomes and data sources

The primary outcomes for efficacy included days of hospitalization and days till clinical improvement. For the secondary outcomes, the proportion of patients who had complete normalization of vital signs, the proportion of patients with total clinical recovery (defined as complete regression of symptoms and persistent symptoms of lower intensity), the proportion of patients with two consecutive negative PCR and the proportion of patients with progressive CT scans were extracted. All patients were classified into two groups, group 1 being the progressive CT group defined by the worsening or advancement of the lung abnormalities visible on repeated CT scans of the chest in COVID-19 patients over time, and group 2 being the non-progressive CT group defined by unchanged and regressive CT scores; the severity level of lung lesions was assessed by Yang et al. “Severity Scoring System“^19^. All these outcomes are compared with the standard of care.

Regarding safety, the primary outcome was comparing the survival probabilities across groups, and the secondary outcome comprised the abnormalities in lab measures.

### Statistical methods

All the categorical data were described by presenting their count and their percentage. Shapiro-Walk test was used to test for the normality of the numerical data assuming the numerical data are not normally distributed if the* p* value was < 0.05.

Descriptive statistics and pairwise comparisons were using the Chi-square test and Fischer exact test for categorical data, and the Wilcoxon rank sum test for numerical data.

Multiple linear and logistic regression were used to account for the potential confounders such as age and gender. The survival analysis employed Cox proportional hazard regression to compare survival probabilities across groups.

## Results

### Demographics and patient characteristics

Our study comprised a total of 310 patients, 98 in arm 1, 64 in arm 2, 75 in arm 3, and 73 in arm 4. The age and gender were comparable between arm 1 and arm 2 as well as between arm 1 and arm 3 [(median age: 36 years old, IQR: 26–50 years old), and (median age: 38 years old, IQR: 27–49 years old), respectively]. However, the age and gender significantly differed between arm 1 and arm 4 (*p* = 0.004 and 0.038 respectively). The study mainly consisted of males ranging from 78.9% to 93.2% across all arms (Table 1).

**Table 1:** Comparison of demographics and efficacy outcomes between arm 2, 3, and 4 in comparison with the standard of care.

Demographic characteristics and efficacy measures		Study arms							
		Standard of care (Arm 1), *N* = 98^1^		Iverzine, Sofosbuvir, Daclatasvir (Arm 2) *N* = 64^1^	Sofosbivir, Ledispavir, Hydroxycholoroquine (Arm 3) *N* = 75^1^			Sofosbuvir, Lidispavir, Ivermectin (Arm 4) *N* = 73^1^	
Age		36 (26, 48)		35 (27, 53)	42 (28, 52)			29 (24, 38)**^2^	
*Gender*									
Female		17 (17.7%)		12 (21.1%)	12 (16.0%)			5 (6.8%)*^2^	
Male		79 (82.3%)		45 (78.9%)	63 (84.0%)			68 (93.2%)	
Days until clinical improvement		5 (4, 7)		5 (4, 7)	5 (4, 8)			6 (5, 8)**^2^	
Days of hospitalization		12 (9, 41)		10 (7, 11)**^2^	11 (8, 14)*^2^			13 (9, 14)	
*Normal vital signs*									
No		56 (57.1%)		38 (59.4%)	48 (64.0%)			54 (74.0%)	
Yes		42 (42.9%)		26 (40.6%)	27 (36.0%)			19 (26.0%)*^2^	
*Total clinical recovery*									
No		98 (100.0%)		59 (92.2%)	75 (100.0%)			70 (95.9%)	
Yes		0 (0.0%)		5 (7.8%)**^2^	–			3 (4.1%)	
*Two consecutive negative PCR*									
No		68 (69.4%)		58 (90.6%)	65 (86.7%)			52 (71.2%)	
Yes		30 (30.6%)		6 (9.4%)**^2^	10 (13.3%)**^2^			21 (28.8%)	
*Progressive CT*									
No	64 (65.3%)		52 (81.2%)			60 (80.0%)	68 (93.2%)		
Yes	34 (34.7%)		12 (18.8%)*^2^			15 (20.0%)*^2^	5 (6.8%)**^2^		

^1^ Median (IQR); n (%).

^2^ Wilcoxon rank sum test; Pearson’s Chi-squared test.

^3^ * P value < 0.05, ** P value < 0.01.

## Outcomes

### Efficacy outcomes

#### Primary outcomes

**Fig. 1:**
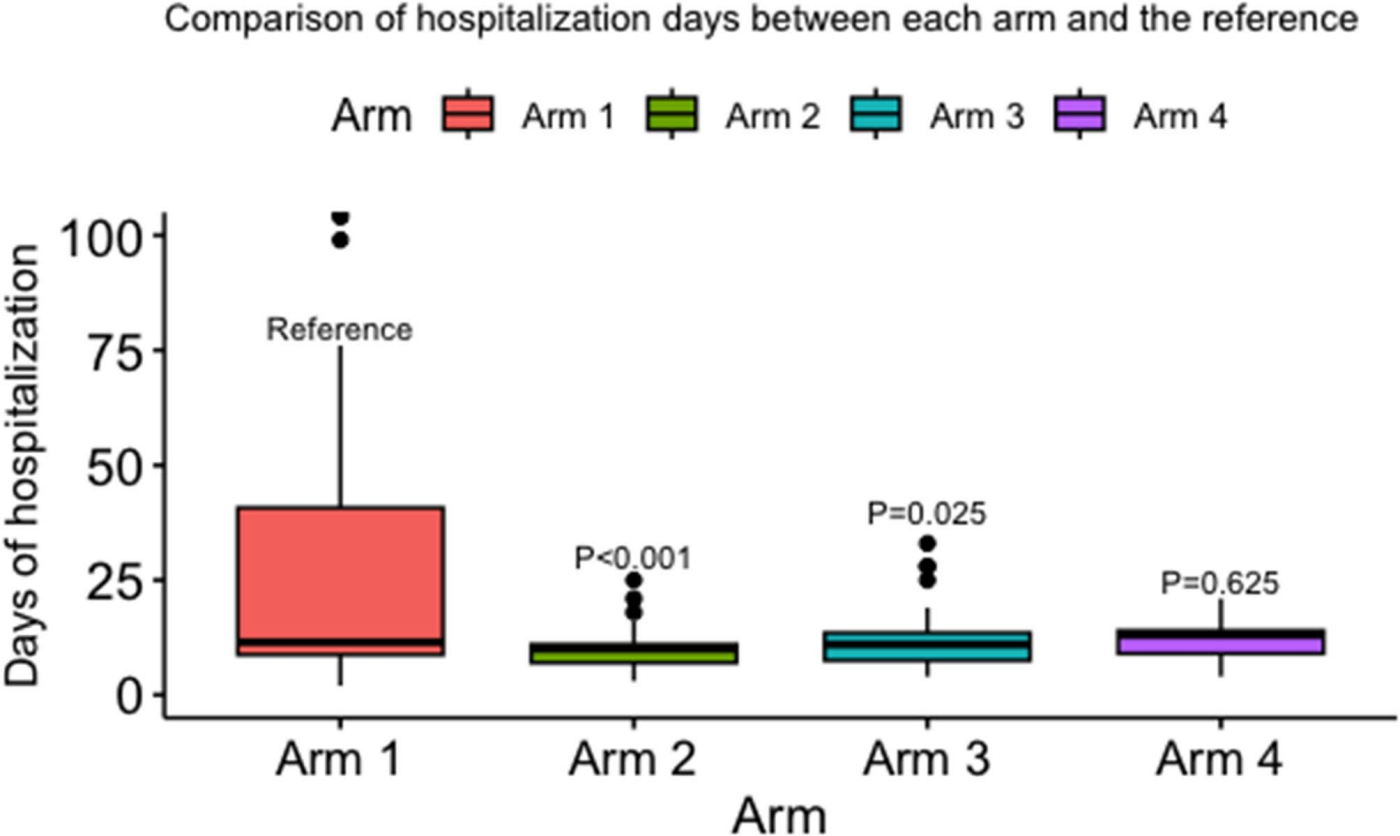
Comparison of hospitalization days between Arm 1 (reference) and the other study arms. Arm 1 = Standard of Care (Hydroxychloroquine and Ivermectin). Arm 2 = Sofosbuvir, Daclatasvir (Sovodak) and Ivermectin. Arm 3 = Sofosbuvir, Ledipasvir (SOF/LED), and Hydroxychloroquine. Arm 4 = Ivermectin and SOF/LED.

The median days of hospitalization were significantly different in arm 1 versus both arm 2 and arm 3 [(median: 12 days, IQR: 9–41 in arm1), (median: 10 days, IQR: 7–11 days in arm 2), (median: 11 days, IQR:8–14 in arm 3), *p* < 0.001and *p* = 0.025, respectively] (Table 1; Fig. 1).

**Fig. 2:**
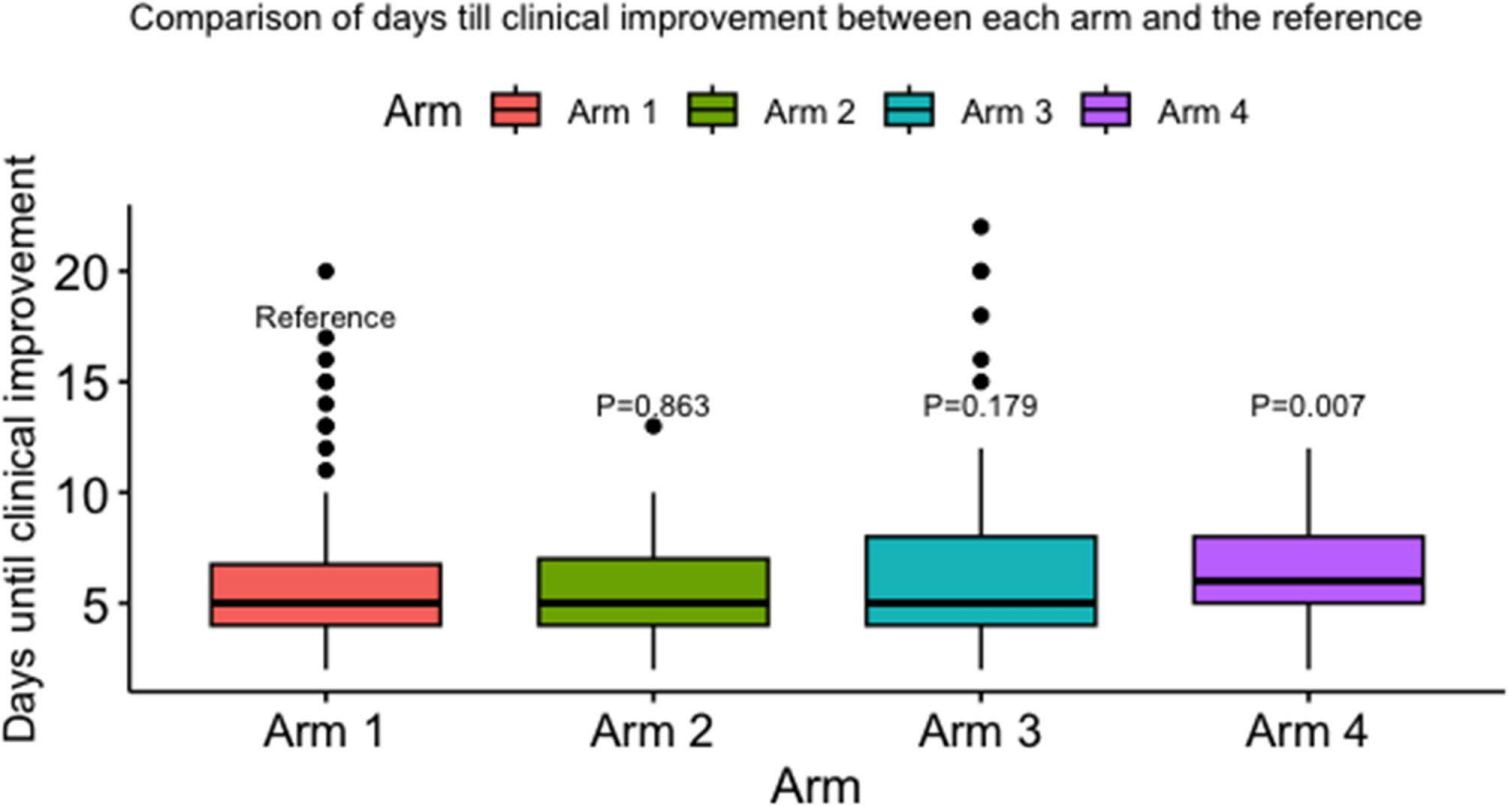
Comparison of days to clinical improvement between Arm 1 (reference) and the other study arms. Arm 1 = Standard of Care (Hydroxychloroquine and Ivermectin). Arm 2 = Sofosbuvir, Daclatasvir (Sovodak) and Ivermectin. Arm 3 = Sofosbuvir, Ledipasvir (SOF/LED), and Hydroxychloroquine. Arm 4 = Ivermectin and SOF/LED.

Regarding the days till clinical improvement, there was a difference between arms 1 and 4 where they were 6 days with an IQR of 5 to 8 days in arm 4 versus a median of 5 days with an IQR from 4 to 7 in arm 1 (*p* = 0.007) (Table 1; Fig. 2).

#### Secondary outcomes

No difference was found in the proportion of people with normal vital signs between arm 1 and arm 2, or between arm 1 and arm 3 (*p* = 0.778 and* p* = −0.361, respectively). However, 26% of the patients had a complete normalization of vital signs in arm 4 versus 43% in arm 1 (*p* = 0.023). Regarding the proportion of patients with total clinical recovery, a statistically significant difference was only found between arms 1 and 2 (*p* = 0.009). Additionally, a statistically significant difference was found between both arm 1 versus arm 2 and arm 1 versus arm 3 in the percentage of patients with two consecutive negative PCR (*p* = 0.001 and *p* = 0.008, respectively) (Table 1). Regarding the proportion of patients with progressive CT scans, all arms displayed a lower and statistically significant difference compared to the standard of care (arm 1). [(34.7% for arm 1, 18.8% for arm 2, 20% for arm 3, and 6.8% for arm 4) and (*p* = 0.028, *p* = 0.034 and *p* < 0.001, respectively)] (Table 1).

### Safety outcomes

The primary safety outcome, comparing the survival probabilities across groups, is discussed below in the regression analyses section.

The secondary outcome comprised the abnormalities in lab measures. Regarding arm 1 versus arm 2, there was a statistical difference in neutrophils and urea measures in the first-time measurements (*p* values = 0.021, 0.004, respectively). Furthermore, the second-time measurements showed a statistical difference in urea and ferritin measures between both groups (*p* value = 0.005, < 0.001, respectively). For the third-time measures, ferritin significantly differed between both groups(*p* value = 0.039) (Table 2).

Regarding arm 1 versus arm 3, there was a statistical difference in hemoglobin, lymphocytes, AST, ferritin, and IL-6 measures in the first-time measurements (*p* value < 0.001, 0.029, 0.044, 0.028, 0.037, respectively). Furthermore, the second-time measurements showed a statistical difference in TLC, lymphocytes, neutrophils, urea, and d-dimer measures between both groups (*p* value = 0.002, < 0.001, 0.006, < 0.001, 0.017, respectively). For the third-time measures, lymphocytes, neutrophils, AST, and urea significantly differed between both groups (*p* value = 0.003, 0.049, 0.046, 0.003, respectively) (Table 2).

Regarding arm 1 versus arm 4, there was a statistical difference in platelet, creatinine, and IL-6 measures in the first-time measurements between both groups (*p* value = 0.028, 0.004, < 0.001 respectively). Furthermore, the third-time measurements showed a statistical difference in TLC, creatinine, and CRP measures between both groups (*p* value = 0.014, 0.022, 0.023, respectively) (Table 2).

**Table 2:** Safety measures compared between study arms.

Safety measures	Study arms			
	Standard of care (reference), *N* = 98^1^	Iverzine, Sofosbuvir, Daclatasvir *N* = 64^1^	Sofosbivir, Ledispavir, HCQ *N* = 75^1^	Sofosbuvir, Lidispavir, Ivermectin, *N* = 73^1^
*First measures*				
HB	14.35 (13.40, 15.30)	14.15 (12.60, 15.15)	13.40 (12.40, 14.30)**	14.75 (13.80, 15.60)
TLC	5.8 (3.9, 7.6)	4.8 (3.9, 6.5)	6.00 (4.55, 7.65)	6.30 (4.40, 7.80)
PLT	196 (163, 243)	210 (170, 265)	199 (164, 263)	229 (179, 272)*
Lymphocytes	29 (22, 40)	33 (24, 44)	23 (15, 37)*	30 (23, 37)
Neutrophils	67 (57, 75)	60 (50, 73)*	71 (56, 80)	66 (58, 71)
ALT	29 (15, 41)	25 (17, 39)	26 (20, 40)	28 (20, 40)
AST	23 (19, 30)	23 (18, 32)	28 (20, 36)*	23 (18, 30)
Urea	24 (21, 32)	31 (25, 40)**	26 (19, 36)	28 (23, 36)
Creatinine	1.00 (0.90, 1.10)	1.00 (0.90, 1.15)	1.00 (0.80, 1.10)	0.90 (0.80, 1.00)**
Ferritin	148 (81, 312)	77 (44, 135)**	354 (122, 813)*	152 (93, 220)
D.Dimer	0 (0, 0)	0 (0, 0)	0.30 (0.13, 0.58)	0.21 (0.09, 0.48)
CRP	19 (5, 62)	20 (7, 35)	25 (11, 56)	15 (7, 47)
IL-6	14 (5, 33)	17 (12, 41)	6 (2, 30)*	3 (2, 12)**
*Second measures*				
HB	14.30 (13.07, 15.30)	14.10 (12.80, 14.95)	13.50 (12.30, 14.67)	14.65 (13.70, 15.60)
TLC	7.0 (5.0, 8.8)	6.7 (5.0, 8.9)	8.7 (6.9, 11.3)*	5.8 (4.4, 9.1)
PLT	244 (199, 290)	260 (190, 308)	255 (210, 298)	258 (218, 308)
Lymphocytes	33 (21, 43)	28 (18, 38)	17 (13, 24)**	33 (22, 44)
Neutrophils	63 (53, 75)	64 (56, 74)	76 (63, 82)**	60 (48, 71)
ALT	42 (25, 71)	40 (25, 63)	44 (24, 81)	44 (25, 99)
AST	26 (19, 44)	33 (19, 46)	28 (17, 34)	29 (20, 47)
Urea	26 (20, 34)	30 (27, 38)**	45 (31, 50)**	28 (21, 34)
Creatinine	0.90 (0.80, 1.10)	1.00 (0.80, 1.15)	1.00 (0.80, 1.10)	0.90 (0.80, 1.00)
Ferritin	173 (107, 261)	72 (46, 100)**	770 (770, 770)	208 (139, 380)
D.Dimer	0 (0, 0)	0 (0, 0)	1 (0, 1)*	0 (0, 0)
CRP	8 (5, 14)	6 (3, 11)	6 (6, 6)	6 (3, 16)
IL-6	16 (2, 54)	17 (5, 28)	19 (3, 103)	15 (2, 104)
*Third measures*				
HB	14.30 (12.90, 15.40)	14.00 (12.40, 15.10)	14.35 (13.40, 15.05)	14.45 (13.40, 15.40)
TLC	8.4 (6.6, 10.9)	7.6 (6.0, 10.2)	11.0 (8.9, 13.1)	6.6 (5.4, 9.4)*
PLT	263 (227, 328)	268 (240, 339)	264 (240, 340)	272 (237, 326)
Lymphocytes	27 (18, 39)	27 (18, 35)	16 (10, 19)**	32 (21, 40)
Neutrophils	68 (58, 77)	66 (59, 75)	78 (72, 84)*	64 (54, 71)
ALT	46 (29, 97)	47 (31, 81)	58 (54, 148)	58 (36, 99)
AST	26 (16, 33)	28 (20, 46)	30 (27, 69)*	31 (19, 40)
Urea	30 (21, 40)	31 (25, 41)	45 (43, 71)**	28 (24, 36)
Creatinine	1.00 (0.90, 1.10)	1.00 (0.90, 1.20)	1.10 (0.90, 1.20)	0.90 (0.80, 1.00)*
Ferritin	114 (67, 192)	71 (50, 109)*	770 (770, 770)	206 (90, 324)
D.Dimer	0.24 (0.17, 0.35)	0.23 (0.20, 0.28)	0.38 (0.38, 0.38)	0.20 (0.14, 0.43)
CRP	7 (4, 11)	4 (3, 8)	162 (162, 162)	3.0 (2.3, 5.6)*
IL-6	59 (7, 286)	13 (9, 40)	64 (32, 303)	210 (170, 255)
*Fourth measures*				
HB	13.60 (12.72, 14.60)	13.55 (13.15, 13.90)	13.80 (13.62, 14.72)	14.20 (13.30, 14.70)
TLC	7.5 (5.4, 12.4)	8.9 (7.1, 11.0)	13.8 (9.8, 14.1)	8.5 (6.3, 11.3)
PLT	248 (196, 273)	226 (202, 252)	212 (186, 230)	284 (218, 330)
Lymphocytes	20 (12, 34)	19 (13, 26)	13 (7, 16)	30 (25, 36)
Neutrophils	76 (68, 85)	75 (69, 80)	79 (75, 84)	62 (52, 71)
ALT	127 (27, 134)	98 (83, 126)	40 (34, 76)	36 (21, 155)
AST	49 (20, 56)	64 (46, 81)	44 (25, 65)	23 (15, 60)
Urea	36 (30, 52)	54 (42, 68)	50 (41, 70)	32 (28, 38)
Creatinine	1.00 (0.90, 1.20)	0.95 (0.78, 1.40)	0.80 (0.75, 0.85)	1.00 (0.98, 1.00)
Death	2 (2.1%)	1 (1.8%)	2 (2.7%)	1 (1.4%)

^1^ n (%); Median (IQR).

^2^ * P value < 0.05 ** P value < 0.01 (Pearson’s Chi-squared test; Wilcoxon rank sum test; Wilcoxon rank sum exact test; Fisher’s exact test).

^3^* HB* hemoglobin,* TLC*  total leukocytic count,* PLT* platelets,* ALT* alanine transaminase,* AST* aspartatetrransaminase,* CRP * c-reactive protein,* IL-6* interleukin-6.

#### Regression analyses

Regarding the primary safety outcome comparing survival probabilities, Cox proportional hazard regression was utilized which showed that the 3 different regimens (arms 2, 3, and 4) had no significant effect on the risk of death, only age if increased by 1 year, will significantly increase the hazard of death by 8% (HR = 1.08, CI = 1.01–1.16, *p* = 0.021) (Supplementary Table S1).

Multiple linear regression was conducted to show the predictors of days till clinical improvement and the days of hospitalization. After adjustment for all other factors, it showed that if age is increased by one year, the days till clinical improvement would increase by 0.06 days when adjusting for all the other factors (beta = 0.06, *p* < 0.001). Additionally, arms 2, 3, and 4 had significantly decreased days of hospitalization by 31, 29, and 29 days respectively compared to arm 1[(beta = − 31, CI = − 43, − 19,* p* value < 0.001), (beta = − 29, CI = − 40, −18,* p* value < 0.001), and (beta = − 29, CI = − 40, − 17,* p* value < 0.001), respectively] (Table 3).

**Table 3:** Multiple linear regression to show the predictors of days till clinical improvement and the days of hospitalization.

	Dependent = Days till clinical improvement			Dependent = Days of hospitalization		
	Beta	95% CI^1^	*p* value	Beta	95% CI^1^	*p* value
*Arm*						
Standard of care	–	–		–	–	
Iverzine, Sofosbuvir, Daclatasvir	−0.85	−1.9, 0.20	0.110	− 31	− 43, − 19	< 0.001
Sofosbivir, Ledispavir, Hydroxycholoroquine	0.64	−0.32, 1.6	0.190	− 29	− 40, − 18	< 0.001
Sofosbuvir, Lidispavir, Ivermectin	0.66	−0.31, 1.6	0.183	− 29	− 40, − 17	< 0.001
Age	0.06	0.03, 0.08	**< 0.001**	− 0.09	− 0.37, 0.18	0.517
*Gender*						
Female	–	–		–	–	
Male	−0.32	−1.4, 0.72	0.543	−5.8	−17, 5.8	0.326

*P* value < 0.001 highly significant, *P* value < 0.05 significant, while none significant if < 0.05.

^1^ *CI*  confidence interval.

Multiple logistic regression was conducted to assess the predictors of the proportion of patients with two consecutive negative PCRs and the normalization of vital signs. After adjustment for all other factors, it showed that arms 2 and 3 had significantly decreased the odds of having two consecutive negative PCR tests compared to the standard of care (*p* = 0.003 & 0.004, respectively) (Table 4).

Regarding vital signs, by increasing age by 1 year, the odds of vital signs normalization will significantly decrease by 2% (OR = 0.98, CI = 0.97–0.99, *p* = 0.037). Additionally, arm 4 had significantly decreased the odds of vital signs normalization by 60% compared to the standard of care (OR = 0.40, CI = 0.20–0.77, *p* = 0.007) (Table 4).

**Table 4:** Multiple logistic regression shows the predictors of the proportion of patients with two consecutive negative PCR and the normalization of vital signs.

	Dependent = Patients with two consecutive negative PCR			Dependent = Normalization of vital signs		
	OR^1^	95% CI^1^	*p* value	OR^1^	95% CI^1^	*p* value
*Arm*						
Standard of care	–	–		–	–	
Iverzine, Sofosbuvir, Daclatasvir	0.26	0.10, 0.61	**0.003**	1.07	0.55, 2.10	0.840
Sofosbivir, Ledispavir, Hydroxycholoroquine	0.32	0.14, 0.68	**0.004**	0.76	0.40, 1.41	0.381
Sofosbuvir, Lidispavir, Ivermectin	0.79	0.40, 1.52	0.477	0.40	0.20, 0.77	0.007
Age	0.98	0.96, 1.00	0.066	0.98	0.97, 0.99	0.037
*Gender*						
Female	–	–		–	–	
Male	0.73	0.35, 1.62	0.425	1.34	0.68, 2.72	0.405

*P* value < 0.001 highly significant, *P* value < 0.05 significant, while none significant if < 0.05.

^1^ *OR*  odds ratio,* CI* confidence interval.

Finally, a logistic regression was conducted to show the predictors of progressive CT scans. After adjustment for all other factors, arms 2, 3 and 4 had significantly decreased odds of progressive CT scan by 61%, 62%, and 85%, respectively compared to arm 1 [(OR = 0.39; CI = 0.17–0.86, *p* value = 0.023), (OR = 0.38; CI = 0.17–0.77,* p* value = 0.010), and (OR = 0.15; CI = 0.05–0.38,* p* value < 0.001), respectively]. Additionally, by increasing age by 1 year, the odds of progressive CT will significantly increase by 4% when adjusting for all the other factors (OR = 1.04, CI = 1.02–1.06, *p* < 0.001) (Table 5).

**Table 5:** Logistic regression to show the predictors of progressive CT scans.

Dependent = progressive CT	OR^1^	95% CI^1^	*p* value
*Arm*			
Standard of care	–	–	
Iverzine, Sofosbuvir, Daclatasvir	0.39	0.17, 0.86	0.023
Sofosbivir, Ledispavir, Hydroxycholoroquine	0.38	0.17, 0.77	0.010
Sofosbuvir, Lidispavir, Ivermectin	0.15	0.05, 0.38	< 0.001
Age	1.04	1.02, 1.06	< 0.001
*Gender*			
Female	–	–	
Male	0.93	0.44, 2.06	0.855

*P* value < 0.001 highly significant, *P* value < 0.05 significant, while none significant if < 0.05.

^1^ *OR* odds ratio,* CI* confidence interval.

## Discussion

Our study tested the safety and efficacy of different antiviral combination regimens in Egyptian adults with moderate COVID-19 infection. The results demonstrated favorable efficacy outcomes for all the treatment arms, where they displayed significantly decreased days of hospitalization by 31, 29, and 29 days, respectively, compared to arm 1 (*p* < 0.001). Regarding the days till clinical improvement, there was a significant difference between arms 1 and 4 [median = 6 days, IQR: 5–8 days in arm 4, versus median = 5 days, IQR: 4–7 in arm 1 (*p* = 0.007)]. Additionally, after regression analysis and adjustment for all other factors, all arms displayed a lower and statistically significant proportion of patients with progressive CT scans by 61%, 62%, and 85%, respectively, compared to arm 1 (*p* = 0.023, *p* = 0.010, and *p* < 0.001, respectively). Finally, all three treatment regimens had no significant effect on the risk of death, nor did they have any significant increases in renal or hepatic lab test results, demonstrating safety. The only variable that significantly increased the risk of death by 8% was increasing age by 1 year (*p* = 0.021).

It is urgently needed to employ early-stage interventions to prevent the infection from worsening and having long-term effects. This is especially important since vaccine hesitation among Egyptians persists despite the country’s efforts to make vaccinations accessible, inexpensive, and appealing to the population^19^. Testing various available antivirals in the Egyptian market is thus relevant and worthy of exploration to prevent disease progression and decrease the death toll. This includes drugs with known antiviral effects or therapy against RNA virus families such as COVID-19, with well-established safety profiles. The ones recruited in our study, SOF/LED and sovodak, are known antivirals with marked availability in the Egyptian pharmaceutical markets^19^ making them easily accessible instead of highly expensive, newly developed drugs.

HCV drugs have been of interest to test as treatments for COVID-19. For instance, sofosbuvir and daclatasvir are FDA-approved for the treatment of chronic HCV. SARS-CoV-2 possesses similar mechanisms of RNA replication as observed in other RNA viruses; subsequently, sofosbuvir and daclatasvir combined were hypothesized to demonstrate efficacy in inhibiting SARS-CoV-2 replication according to in vitro and in silico research^12,20^. Other repurposed combination HCV antivirals include sofosbuvir/ledipasvir (SOF/LED) and protease inhibitors, which have also been tested against COVID-19 in multiple studies^9,12,13,20,21^. These different combinations and approaches drove our study to compare the most effective ones in the unique Egyptian population.

Sovodak (sofosbuvir/daclatasvir) has been tested in multiple clinical trials for its effect on clinical recovery. A recent meta-analysis of three Iranian trials showed that it improved the time to clinical recovery [HR = 2.04 (95% CI = 1.25–3.32), *p* = 0.004] and exhibited lower days of hospitalization compared to the control group [MD = − 0.56 (95% CI = − 0.86 to − 0.26)]^12^. This is comparable to our results where the proportion of patients with total clinical recovery was significantly higher in the sovodak arm compared to the standard of care (*p* = 0.009), and the median days of hospitalization days were also lower in the sovodak arm (*p* < 0.001). This should be cautiously interpreted while considering the drug combination used (alone versus with ivermectin in our case) and the difference in patient count where they comprised 64 in our study arm compared to 92 patients in the meta-analysis.

A multicenter, randomized controlled clinical trial in adults with moderate or severe COVID-19 admitted to four university hospitals in Iran randomized patients into a treatment arm receiving sofosbuvir and daclatasvir plus standard care, or a control arm receiving standard care alone. The clinical recovery within 14 days was achieved by 88% in the treatment arm versus 67% in the control arm (*P* = 0.076), where they also showed a significantly shorter median duration of hospitalization [6 days (IQR 4–8)] than the control group [8 days (IQR 5–13)] (*p* = 0.029)^22^. Furthermore, Abbass et al. compared sofosbuvir/daclatasvir to the standard of care, with all patients receiving additional therapies, such as HCQ and ivermectin, according to the treating physician’s clinical judgment. Patients receiving sofosbuvir/daclatasvir showed significant clinical improvement compared to standard of care on both day 7 (*p* = 0.041) and day 10 (*p* = 0.040), while no significant differences in mortality were observed (*p* = 0.329)^23^. Our finding similarly showed the median days of hospitalization were significantly different in arm 1 (control) versus arm 2 (sofosbuvir/ daclatasvir plus ivermectin) [(median: 12 days, IQR: 9–41 in arm1) versus (median: 10 days, IQR: 7–11 days in arm 2), *p* < 0.001], where arm 2, had significantly decreased days of hospitalization by 31, days compared to arm 1 [(beta = − 31, CI = −43, −19,* p* value < 0.001). Additionally, there were no statistical differences in mortality rates (2.1% for arm 1 versus 1.8% for arm 2). This agreement highlights the favorable efficacy of sofosbuvir/ daclatasvir combined with either ivermectin or HCQ in enhancing COVID-19 patients’ outcomes.

Khalili et al. compared SOF/LED to the standard of care (HCQ and atazanavir/ritonavir). They found no significant differences in the incidence of clinical improvement (*p* = 0.65), length of hospital stay (*p* = 0.98), or 14 day mortality (*p* = 0.60) between the groups. However, the SOF/LED arm had a shorter time to clinical improvement (2 [1–3.75]) than the control group (4 [2–5, *p* = 0.02)^14^. In contrast, our findings for arm 3, which utilized SOF/LED with HCQ, showed significant and shorter median days of hospitalization compared to arm 1(*p* = 0.025). Additionally, there was no difference between both arms in terms of time to clinical improvement. These disparities could be attributed to the difference in study design; ours is a retrospective cohort compared to Khalili’s randomized controlled trial design. Moreover, we used SOF/LED in combination with HCQ, while their method included atazanavir/ritonavir. Finally, their patient count was 42 versus 75 in our study, all of which may account for differences in results and significance.

COVID-19 has been identified to cause alterations in lab measures, including increased neutrophil count and platelet-lymphocyte ratio, which are linked to a worse clinical outcome^24^. Additionally, it is considered a risk factor for elevations in urea, creatinine, D-dimer, ALT, and AST levels^3^. Thus, monitoring these parameters can be used as an indicator of the efficacy of our tested treatment regimens, where we found multiple results in favor of these repurposed antiviral combinations versus the standard of care. For instance, arm 2 displayed significantly lower ferritin levels throughout all the lab measures compared to arm 1, arm 3 showed lower lymphocytes compared to arm 1, and arm 4 showed significantly lower creatinine levels than arm 1 in both 1st and 3rd measures, and lower CRP in 3rd measures. Furthermore, the tested treatments did not lead to any significant increases in renal or hepatic lab test results, thus indicating the tolerability of these combined treatments. In real-world clinical practice, not all lab measures are ideal, however, these results demonstrate promising efficacy and safety outcomes for these treatment regimens in COVID-19 patients.

Similar contexts testing the efficacy of age and other variables on mortality have been present in other studies. For instance, a recent study of multiple antiviral regimens including remdesivir and favipiravir showed that age was significantly correlated with the overall fatal cases (*p* < 0.001). Additionally, multivariate analysis of variance revealed that age significantly affected the mortality rate of patients(*p* > 0.001)^25^. This is similar to our findings where age if increased by 1 year, will significantly increase the death risk by 8% (*p* = 0.021). However, their population mainly consisted of severe COVID-19 cases, which could justify the need for different forms of antivirals, especially remdesivir, which is the first FDA-approved treatment for COVID-19 use in emergency cases^26^.

Our work is distinctive in that it provides a thorough assessment of whether or not progressive changes in CT scans occur with various antiviral treatment regimens. The evaluation of radiographic disease progression has gotten comparatively less attention than clinical outcomes, such as time to clinical improvement or mortality, which have been the main focus of previous research. Our results demonstrated that all arms displayed lower and statistically significant odds of progressive CT scan compared to arm 1 (*p* = 0.023, *p* = 0.010, *p* < 0.001, respectively). Additionally, by increasing age by 1 year, the odds of progressive CT will significantly increase by 4% when adjusting for all the other factors (*p* < 0.001). This suggests that our proposed antiviral treatment combinations may have a more favorable effect on limiting the radiographic progression of the disease. These findings are particularly important as in our previous study, the extent of lung involvement and the rate at which the disease progresses were weighted from the most significant independent predictors of mortality when developing and validating our “EGY.Score” for predicting COVID-19 hospitalized patients mortality^27^, which have a major impact on patient management and long-term results. Incorporating this radiographic objective can aid healthcare professionals in making the best treatment decisions for patients with moderate COVID-19.

Though the aim was to test new treatment combinations, we noticed that arm 1 comprising HCQ plus ivermectin, displayed a significant and shorter number of days till clinical improvement compared to arm 4 (*p* = 0.007). Additionally, it demonstrated the highest percentage of complete normalization of vital signs (*p* = 0.023). This shows that despite the many other benefits of novel combinations, the standard of care remains a very good option for moderate COVID-19 patients.

### Limitations and strengths

Our study has some potential limitations. For instance, due to the retrospective nature, we couldn’t identify or present treatment-related side effects in the safety outcomes as they are better monitored in an RCT study. Additionally, our study’s end date was in 2022, after which new antivirals as molnupiravir or remdesivir have been added to the national guidelines. Furthermore, the single-center design and relatively small sample sizes are limitations that should be considered when interpreting the findings. Finally, the baseline differences in some groups (like the relatively younger age in arm 4), limit the conclusions that can be drawn from our study.

On the other hand, the strengths of this study include the comprehensive assessment of both safety and efficacy endpoints, as well as the inclusion of a well-defined reference group for comparison. Moreover, these repurposed antiviral combination regimens’ relative safety and efficacy outcomes demonstrate successful and potential drug candidates that can be utilized for COVID-19 treatment and its emerging variants. Additionally, our study is unique in identifying CT improvement associated with the anti-HCV antiviral regimen which can help guide future case-specific treatment approaches.

## Conclusion and future directions

In conclusion, our research sheds light on how well various antiviral combinations perform in actual COVID-19 clinical settings. The results demonstrate that days of hospitalisation were significantly lower in arms 2 and 3 compared to the standard of care. Additionally, all treatment arms displayed a lower and statistically significant difference in progressive CT scans compared to the standard of care. Finally, most lab values were comparable, and none of the tested arms had a significant effect on the risk of death. This demonstrates their efficacy and safety as potential treatment options for moderate COVID-19 patients, where early identification and treatment can prevent disease progression and complications.

However, to properly verify the safety and effectiveness of these therapeutic techniques, more comprehensive and carefully planned clinical trials are required. Incorporating newly approved antivirals in the trials can be beneficial to check their clinical response (alone or in combinations) in the Egyptian population while keeping in mind the selection of generics of appropriate cost to the public and available in the country’s pharmaceutical market.

## Supplementary Information

Below is the link to the electronic supplementary material.

Supplementary Material 1

## Data Availability

All data generated or analyzed during this study are included in this published article.
