# Supplementary material for: Safety and efficacy of different therapeutic regimens in Egyptian adults with moderate COVID-19 infection (EVEREST): a real-world retrospective study
Source: Sci Rep. 2025 Oct 20;15:36477. doi: 10.1038/s41598-025-23660-1 (PMC12537978; doi:10.1038/s41598-025-23660-1)
Supplement: Supplementary file 1 — Supplementary Material 1 [file 41598_2025_23660_MOESM1_ESM.docx]

**Supplementary Table S1. Cox proportional hazard regression to show the predictors of the risk of death:**

| Dependent= risk of death | HR*^1^* | 95% CI*^1^* | p-value |
| --- | --- | --- | --- |
| Arm |  |  |  |
| Standard of care | — | — |  |
| Iverzine, Sofosbuvir, Daclatasvir | 3.46 | 0.27, 43.9 | 0.338 |
| Sofosbivir, Ledispavir, Hydroxycholoroquine | 6.13 | 0.46, 81.2 | 0.169 |
| Sofosbuvir, Lidispavir,  Ivermectin | 2.32 | 0.17, 31.3 | 0.525 |
| Age | 1.08 | 1.01, 1.16 | **0.021** |
| Gender |  |  |  |
| Female | — | — |  |
| Male | 0.08 | 0.01, 0.81 | **0.032** |
| *^1^* HR = Hazard Ratio, CI = Confidence Interval | | | |
